# AWP1 Restrains the Aggressive Behavior of Breast Cancer Cells Induced by TNF-α

**DOI:** 10.3389/fonc.2021.631469

**Published:** 2021-03-18

**Authors:** Eun-Young Kim, Ji-Eun Kim, Bongkun Choi, Jiyeon Kweon, Si-On Park, Hee-Seop Lee, Eun-Jin Lee, Soyoon Oh, Ha Rim Shin, Hyuksu Choi, Yongsub Kim, Eun-Ju Chang

**Affiliations:** ^1^ Department of Biomedical Sciences, Asan Medical Center, University of Ulsan College of Medicine, Seoul, South Korea; ^2^ Stem Cell Immunomodulation Research Center, Asan Medical Center, University of Ulsan College of Medicine, Seoul, South Korea; ^3^ Department of Biochemistry and Molecular Biology, Asan Medical Center, University of Ulsan College of Medicine, Seoul, South Korea

**Keywords:** AWP1, breast cancer, migration, TNF-α, NF-κB, epithelial–mesenchymal transition, reactive oxygen species

## Abstract

TNF-α plays a crucial role in cancer initiation and progression by enhancing cancer cell proliferation, survival, and migration. Even though the known functional role of AWP1 (zinc finger AN1 type-6, ZFAND6) is as a key mediator of TNF-α signaling, its potential role in the TNF-α-dependent responses of cancer cells remains unclear. In our current study, we found that an AWP1 knockdown using short hairpin RNAs increases the migratory potential of non-aggressive MCF-7 breast cancer cells with no significant alteration of their proliferation in response to TNF-α. A CRISPR/Cas9-mediated AWP1 knockout in MCF-7 cells led to mesenchymal cell type morphological changes and an accelerated motility. TNF-α administration further increased this migratory capacity of these AWP1-depleted cells through the activation of NF-κB accompanied by increased epithelial-mesenchymal transition-related gene expression. In particular, an AWP1 depletion augmented the expression of Nox1, reactive oxygen species (ROS) generating enzymes, and ROS levels and subsequently promoted the migratory potential of MCF-7 cells mediated by TNF-α. These TNF-α-mediated increases in the chemotactic migration of AWP1 knockout cells were completely abrogated by an NF-κB inhibitor and a ROS scavenger. Our results suggest that a loss-of-function of AWP1 alters the TNF-α response of non-aggressive breast cancer cells by potentiating ROS-dependent NF-κB activation.

## Introduction

Tumor necrosis factor α (TNF-α) is a potent proinflammatory cytokine that regulates inflammatory processes during tumor development and promotes tumor progression by enhancing cancer cell proliferation, survival, and migration (1–3). In general, it is well established that the chronic production of TNF-α in a tumor microenvironment contributes to malignant tumor progression by mediating tumor/stromal cell interactions, inducing a range of cytokines, and causing direct DNA damage (1, 4). For instance, TNF-α induces an epithelial-mesenchymal transition (EMT) that facilitates cancer cell migration in colon cancer (5), prostate cancer (6) and breast cancer (7) by contributing to cancer initiation and progression. This cytokine promotes inflammation-associated cancer progression *via* activating nuclear factor-κB (NF-κB) signaling (8). However, certain breast cancer patients display resistance to TNF-α responses (9). Indeed, non-aggressive MCF-7 and aggressive MDA-MB-231 breast cancer cells (10) show diverse tumor behaviors in response to TNF-α, including cancer cell proliferation and migration to the TNF-α-induced tumor response (11–13) due to discrepancies in TNF-α-mediated NF-κB activation (14, 15). This NF-κB/TNF-α positive feedback loop has been suggested to contribute to the aggressiveness of the tumor leading to the expression of several pro-survival genes (16), thereby further promoting tumor progression (17). Since elevated TNF-α in aggressive breast cancers has been linked to lymph node metastasis (18), it is believed that the elevation of TNF-α is associated with aggressive clinical behavior and a poorer prognosis in malignant cancers (19).

TNF-α is regarded as a potent mutagen that promotes cellular transformation through the induction of reactive oxygen species (ROS) (20), which can affect cancer cell migration/invasion and thereby promote cancer initiation and progression (21–23). The cumulative evidence to date suggests that an increased intracellular ROS level can alter the rates of cell migration/invasion by directly regulating actin cytoskeleton reorganization and modulating several pathways, namely NF-κB, integrins, small GTPases, MAPK, PI3-K, FAK, AP-1, and matrix metalloproteinases (MMPs) (24–26). Of note in particular, TNF-α induced-ROS generation leads to DNA damage and increases malignant transformation through the activation of NF-κB (20). This supports a promoting effect of the TNF-α/NF-κB/ROS axis on tumor progression.

We previously identified that AWP1 (zinc finger AN1 type-6, ZFAND6), an adaptor protein, interacts with tumor necrosis factor receptor-associated factor 2 (TRAF2) to regulate NF-kB activation in response to TNF-α (27). With regard to the effect of AWP1 on NF-kB activity, a previous genome-wide survey suggested that AWP1 is a potent inhibitor of NF-κB activation (28). In addition, Fenner et al., identified AWP1 as a novel polyubiquitin binder that regulates NF-κB activity (29). These findings suggested the possibility that AWP1 may regulate NF-κB signaling and thereby modulate TNF-α-mediated responses in cancer cells. However, the potential involvement of AWP1 in cancer progression in response to TNF-α is still unknown.

In our present study, we investigated the potential role of the AWP1 protein in breast cancer cells in response to TNF-α. Our results suggest that AWP1 may act as an intracellular regulator of TNF-α-mediated cancer cell responses by regulating ROS/NF-κB activation.

## Materials and Methods

### Cell Cultures

The breast cancer cell lines, MCF-7, T47D, MDA-MB 231 (ATCC, Manassas, VA), and MCF-7/AWP1 KO cells were cultured in DMEM (Hyclone, GE Healthcare Life Sciences), supplemented with 10% fetal bovine serum (FBS), in a humidified atmosphere at 37°C with 5% CO_2_.

### CRISPR-Cas9-Mediated Gene Knockout in MCF-7 Cells and Establishment of Knockout Cell Lines

We cloned AWP1 target gRNAs into the lentiGuide-puro (addgene # 52963) vector in accordance with the recommended protocol. Briefly, annealed oligonucleotides for each gRNA (5’-caccgAGGTGTGCAACTGAGGAACA-3’, 5’-aaacTGTTCCTCAGTTGCACACCTc for AWP1 #1 gRNA, and 5’-caccgAATGGTAGAATAAGCCCACC-3’, 5’-aaac GGTGGGCTTATTCTACCATTc-3’ for AWP1 #2 gRNA) were ligated into the BsmbI-digested lentiGuide-puro vector and the resulting plasmid DNA was confirmed by Sanger sequencing.

To knockout the AWP1 gene in MCF-7 cells, 10^5^ cells were seeded into the wells of a 24-well plate one day before transfection, and a total of 1 μg of plasmid DNA [100 ng of pMAX (Lonza), 300 ng of p3s-Cas9HC (addgene #43945), and 300ng of each gRNA] was transfected using Lipofectamine 2000, in accordance with the manufacturer’s protocol. The transfected cells were selected with 1 μg/ml puromycin 24 h after transfection for two days. To confirm the initial mutation frequencies, genomic DNA was extracted from the selected cells and analyzed by targeted deep sequencing.

To isolate single clones, the selected cells were seeded into 96-well plates at a 0.3 cell/well density and maintained for two weeks. Single clones were picked and divided into two sets, one for analysis and one for maintenance. Cell lysates from single clones were PCR analyzed by targeted deep sequencing to validate the knockout of AWP1. The primer sequences used are listed in **Table S3**.

### Western Blotting

For western blotting analysis, cell lysates obtained from MCF-7 breast cancer cells were resolved by sodium dodecyl sulfate–polyacrylamide gel electrophoresis (SDS-PAGE) and electrophoretically transferred to a polyvinylidene difluoride membrane (Bio-Rad). Non-specific interactions were blocked using 5% bovine serum albumin solution in Tris–buffered saline (20 mM Tris/HCl, pH 7.6, 150 mM NaCl, and 0.1% Triton X-100) for 1 h. The membranes were then incubated with anti-AWP1, Snail (Novus Biologicals, Centennial, CO), IL-6 (Santa Cruz Biotechnology, Santa Cruz, CA), IL-8 (Abcam, Cambridge, MA), Slug (Cell Signaling Technology Inc., MA), and β-actin (Sigma, St. Louis, MO) antibodies for 1 h at room temperature (RT). Membranes were then incubated with the appropriate secondary antibodies conjugated with HRP and immunoreactivity was detected by the use of an enhanced chemiluminescence detection kit (Millipore, Billerica, MA).

### Transwell Assay

Cell chemotaxis was assessed as described previously (30). 5 × 10^4^ cells of MCF-7, MCF-7/AWP1 KO, MDA-MB231, or T47D cells were loaded into the inner membrane of the upper chamber of a 8-μm pore size Transwell system (Costar, Corning, NY). To evaluate its chemotactic potential, TNF-α (20 ng/ml, R&D systems, Minneapolis, MN) was then added to the lower chamber. After incubation for 24 h to allow for migration, cells that had not migrated were removed from the top of the membrane with a cotton swab. The migrated cancer cells in the outer membrane of the upper chamber were then stained with hematoxylin and counted under a microscope.

### Cell Proliferation and Clonogenic Cell Growth Assay

Breast cancer cells (each 1×10^3^ cells) were counted and added to 96-well plates and cultured for 24, 48, and 72 h. Next, 10 μl of Cell Counting Kit (CCK-8) solution (Dojindo, Kumamoto, Japan) was added to each well at each time point. The cells were cultured with CCK-8 for 3 hours, and then the absorbance at 450 nm was measured using the BioTek ELX800 system (BioTek, Winooski, VT).

### Wound-Healing Assay

MCF-7 or MDA-MB231 cells (5 x 10^5^) were cultured to confluence in 6-well plates, and two separated scratch wounds were made in each well using a sterile 200 μl pipette tip. Cells lifted in the process of scratching were gently removed by washing in PBS, and fresh growth medium was then added. After loading the cells with CellTracker™ Green (Thermo Fisher Scientific, Waltham, MA), cells were photographed under a light microscope after 24 h of incubation. The migration area was calculated using image J software.

### Reverse Transcription–Polymerase Chain Reaction (RT-PCR) and Quantitative-PCR (qPCR)

Total RNA was extracted from cultured breast cancer cells using Trizol reagent (Invitrogen Life Technologies) following the manufacturer’s instructions. A RevertAid First strand cDNA Synthesis kit (Thermo Fisher Scientific) was used to synthesize cDNA from RNA and subsequent PCR was performed in a T100™-Thermal Cycler (Bio-Rad, Hercules, CA). The resulting PCR products were analyzed by electrophoresis in a 2% agarose gel and imaged using an ultraviolet gel imaging system (Bio-Rad). qPCR analysis was performed in optical 96-well plates using SYBR Green PCR master mix (Roche, Penzberg, Germany) and the Light Cycler 480 Real-time PCR Detection System (Roche), in accordance with the manufacturer’s instructions. The primers used are described in Supporting Information **Table S1**. Gene expression was normalized to that of a GAPDH internal control. The relative expression of the target genes was calculated by the standard curve method using the target Ct values and the Ct value for GAPDH.

### Generation of Recombinant Lentivirus Expressing AWP1 and Transduction

*AWP1* gene was amplified with RT-PCR using specific primers: forward primer 5’-atggctcaagaaactaatcaca-3’ and reverse primer 5’- tcaaatcttttggatcttttcacc-3’, which contained Xbal and Bam H1 sites, respectively. The PCR product was subcloned into the lentiviral vector pCDH-CMV-MCS-EF1-Puro (System Biosciences, Mountain View, CA, USA) to generate pCDH-AWP1. Then, the lentiviral AWP1 plasmid was co-transfected into 293FT cells (Invitrogen, USA) along with different packaging plasmids, PAX2 and MD2 to generate lentivirus. For lentivirus transduction, MCF-7 and MDA-MB231 cells were cultured and infected at a multiplicity of infection (MOI) of 10 with either lentivirus containing the AWP1 gene (pCDH-AWP1) or empty virus (EV) containing 8 μg/ml of polybrene according to the manufacturer’s instructions. Western blotting was performed to evaluate AWP1 expression in the MDA-MB231 cells infected with pCDH-AWP1.

### Lentiviral shRNA Transduction

The target sequences of the lentiviral shRNA vectors (GeneCopoeia) were as follows: negative control shRNA: 5’-TG GCTGCATGCTATGTTGA-3’; AWP1 shRNA #4, 5’-GCACGGGAGAATATACAAATA-3’; AWP1 shRNA #5, 5’-GGATCCACAAGTCCTTGTTCC-3’ (shRNAs targeting different regions of the human AWP1 transcript (NM_000584.2)). In brief, shRNA expression plasmids and HIV based packaging plasmids (containing genes: Gag/Pol, Rev, VSV-G) were mixed with Lipofectamine 3000 (Invitrogen) reagent in Opti-MEM (Invitrogen) to transfect 293FT cells. The culture medium was replaced with fresh DMEM supplemented with 10% fetal bovine serum (FBS) within 48 h post-transfection. The lentivirus-containing culture medium was collected, aliquoted, and stored at -80°C. MCF-7 cells were transduced with lentiviral media at a multiplicity of infection of 5. The AWP1 knockdown was confirmed by qPCR and western blotting.

### Luciferase Reporter Gene Assays for NF-κB Promoter Activity

MCF-7 WT, MCF-7 AWP1 knockout (KO), or MDA-MB231 cells were transiently transfected with the pNF-κB-Luc reporter plasmid, cultured for 24 h, and then serum-starved for 12 h. The transfected cells were then stimulated with 20 ng/ml TNF-α for the indicated times, collected, and then assayed using a Dual-Luciferase Assay kit (Promega. Madison, WI). Firefly luciferase activities driven by a specific promoter were normalized in each case to respective *Renilla* luciferase activity driven by the TK promoter as a control for transfection efficiency.

### Measurements of Reactive Oxygen Species (ROS) by Flow Cytometry

AWP1 WT or AWP1 KO cells were plated in 6-well plates and incubated for 24 h in DMEM supplemented with 10% FBS and ROS levels were measured as described previously (31). In brief, cells were incubated with H_2_O_2_-sensitive fluorophore 2’, 7’-dichlorofluorescein diacetate (10 μM DCF-DA; Molecular Probes) for 30 min in the dark. For ROS inhibition, the cells were pretreated with N-acetyl-L-cysteine (NAC) for 30 min prior to TNF-α stimulation. DCF fluorescence was measured using a FACSCalibur flow cytometer (BD Biosciences). Values represent means ± SD of DCF fluorescence from three independent experiments.

### Statistics

**Figure 1:**
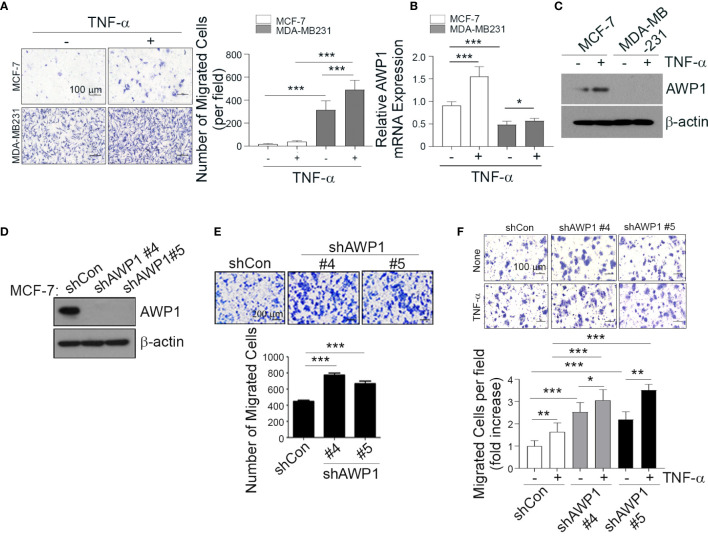
AWP1 knockdown in MCF-7 breast cancer cells enhances TNF-α-induced responses. **(A)** MDA-MB231 and MCF-7 cells were loaded into the upper chamber of a Transwell system and stimulated with 20 ng/ml TNF-α for 24 h in the lower chamber. The migrated cells were stained with hematoxylin (left) and the number of migrated cells was quantified (right); ***p < 0.001. **(B, C)** TNF-α strongly induces AWP1 mRNA expression in MCF-7 cells compared to MDA-MB231 cells. Both cell types were incubated with or without 20 ng/ml TNF-α for 24 h and AWP1 mRNA and protein levels were assayed by qPCR **(B)** and western blotting **(C)**, respectively; *p < 0.05, ***p < 0.001 vs the indicated group. **(D, E)** AWP1 knockdown increases the migration ability of MCF-7 cells. Cells were transfected with control shRNA or the AWP1-specific shRNAs, #4 and #5. The protein knockdown of AWP1 was confirmed by western blot **(D)** and the migratory capacity of the cells was monitored using a Transwell migration assay at 24 h after transfection **(E)**. ***p < 0.001 versus control shRNA. **(F)** AWP1 knockdown enhances TNF-α-induced chemotactic cell motility. MCF-7 cells transfected with control shRNA or AWP1-specific shRNAs were loaded into the upper chamber of the Transwell system and stimulated with 20 ng/ml TNF-α for 24 h in the lower chamber. The number of migrated cells was then was stained (*upper*) and quantified (*lower*); *p < 0.05, **p < 0.005, or ***p < 0.001 versus the shRNA control. All statistical comparisons were performed using t-tests (two groups) or ANOVA with a Tukey post-test (multiple groups).

**Figure 2:**
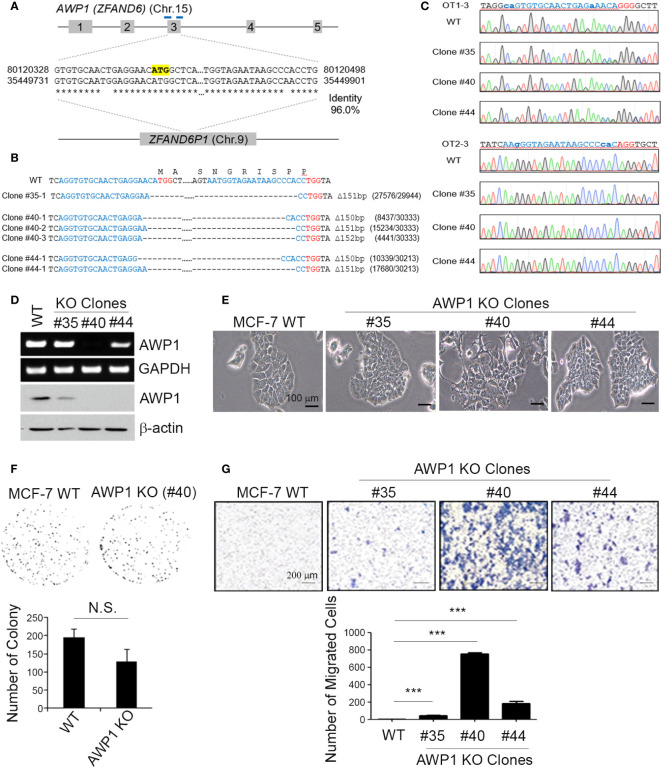
A CRISPR/Cas9-mediated AWP1 knockout in MCF-7 breast cancer cells induces morphological changes and augments their migratory capacity. **(A–C)** Establishment of *AWP1* knockout MCF-7 cell lines. The *AWP1* gene has a pseudogene, ZFAND61, which has a 96% identity with *AWP1* at exon 3. To avoid potential off-target effects, two gRNAs with target sequences that contain exon-intron junctions were selected to delete exon 3. The target sites of these gRNAs are indicated in the blue bar and the start codon of *AWP1* is highlighted in yellow **(A)**. The target sites of three single clones were confirmed by targeted deep-sequencing. The nucleotide sequences and amino acid sequences are shown together. The 20-nt target sequences and PAM are denoted in blue and red, respectively. The read counts of each mutated allele are listed next to the corresponding sequences **(B)**. The Sanger sequencing results of representative potential off-target sites of each gRNA are shown. The 20-nt mismatched-target sequences and PAM are highlighted in blue and red, respectively. The mismatched nucleotide sequences are indicated in lowercase **(C)**. **(D)** Validation of the AWP1 knockout (KO) in MCF-7 cells. Subconfluent MCF-7 control wild-type (WT; CRISPR-Cas9 mediated control group) and MCF-7 AWP1 KO clones (#35, #40, or #44 clones of CRISPR-Cas9 mediated AWP1 knockout group) were harvested and the AWP1 mRNA and protein expression levels were determined utilizing RT-PCR and western blotting analysis, respectively. **(E)** An AWP1 knockout in MCF-7 cells induces morphological changes. Images of growing cells were captured at a 20X magnification using an inverted light microscope. **(F)** AWP1 KO MCF-7 cells display no changes in their clonogenic ability. N.S., not significant. **(G)** Migration was significantly augmented after a stable AWP1 knockout in MCF-7 cells, as detected by Transwell migration assay. ***p < 0.001 relative to the control group using an independent sample t-test. All statistical comparisons were performed using t-tests (two groups) or ANOVA with a Tukey post-test (multiple groups).

**Figure 3:**
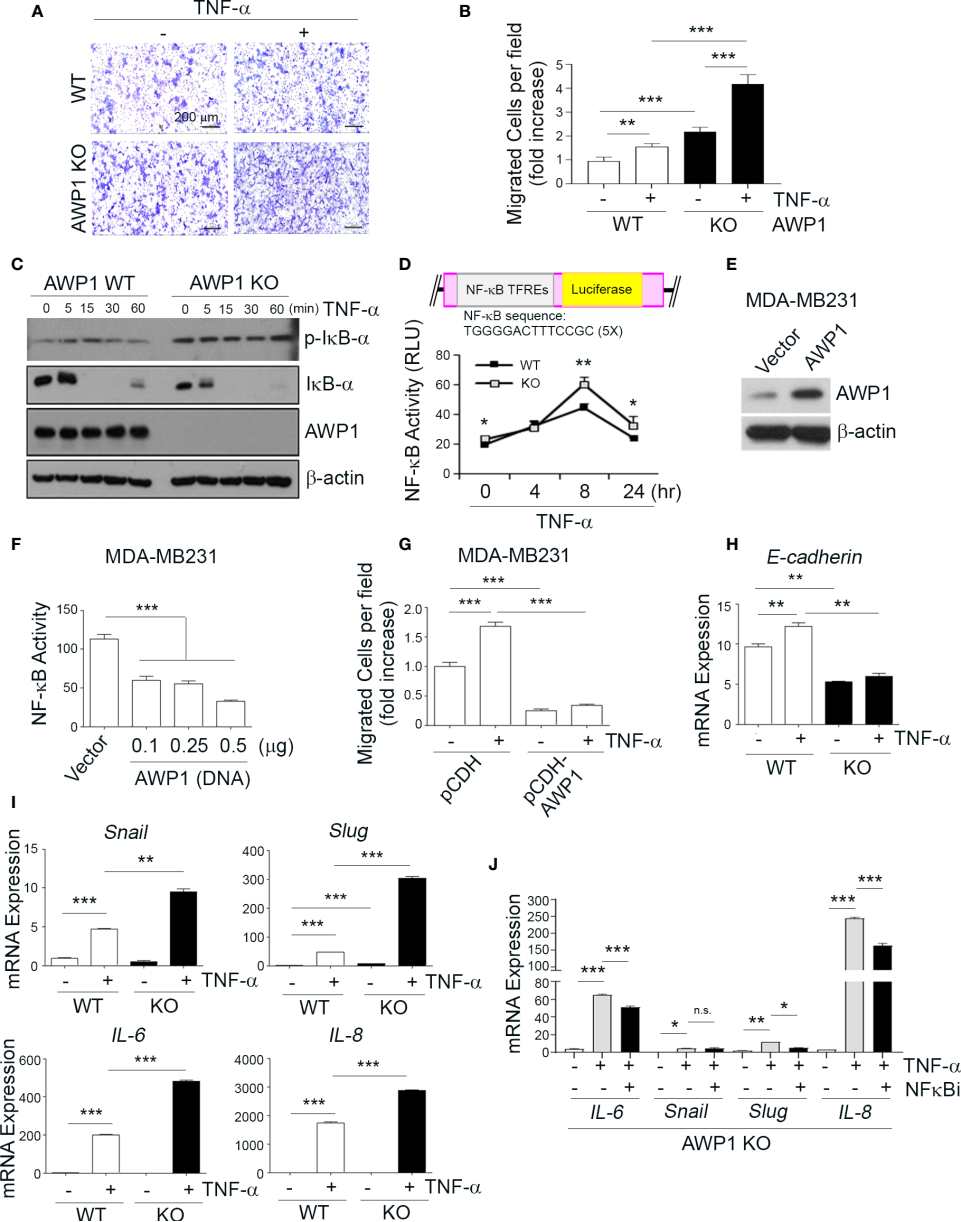
AWP1 depletion in MCF-7 breast cancer cells promotes NF-κB activity and TNF-α-induced migration ability. **(A, B)** An AWP1 knockout enhances the chemotactic migratory potential of MCF-7 cells induced by TNF-α stimulation. WT or AWP1 KO MCF-7 cells were plated onto Transwell inserts and incubated with TNF-α (20ng/ml) in the lower chamber to induce migration. Migrated cells were fixed, stained, and photographed at 24 h after plating **(A)**, and the number of migrated cells was quantified **(B)**. Quantitative data are shown as bar graphs with each data point representing duplicate analyses in performed in parallel that were repeated three times (n = 6). Values are means ± SEM; **p < 0.005, ***p < 0.001 versus the indicated group. **(C, D)** An AWP1 deficiency augments NF-κB activity after TNF-α treatment. WT or AWP1 KO MCF-7 cells were stimulated with 20 ng/ml TNF-α for the indicated times. The protein expression of phospho-IκB-α, IκB-α, AWP1, and β-actin was analyzed by immunoblotting **(C)** and NF-κB promoter activity was determined in WT or AWP1 KO MCF-7 cells **(D)**; *p < 0.05, **p < 0.005 vs the corresponding control. TFREs indicates transcription factor response elements. Luciferase assay was performed at least three times independently in triplicate. **(E)** AWP1 overexpression in MDA-MB231 breast cancer cells was confirmed by immunoblot assay. **(F)** Overexpression of AWP1 attenuates NF-κB activity of MDA-MB231 cells. Cells were transiently transfected with or without vector containing wild-type AWP1 and the NF-κB promoter-luciferase reporter plasmid as indicated and the luciferase activity in the extracts was measured using a Dual luciferase assay system as described materials and methods. ***p < 0.001 vs control vector. **(G)** AWP1 overexpression suppresses TNF-α-induced chemotactic migration potential of MDA-MB231 cells. MDA-MB231 cells were infected with lentiviruses harboring a control vector (pCDH) or with a AWP1 construct (pCDH-AWP1). Two days after transfection, mock and AWP1 overexpressed cells were loaded into the upper chamber of the Transwell system and stimulated with 20 ng/ml TNF-α for 8h in the lower chamber. The migrated cells were stained and quantified by Tukey test. ***p < 0.001 versus corresponding group. **(H, I)** AWP1 depletion in MCF-7 cells dramatically increases the expression of epithelial-mesenchymal transition (EMT)-related genes after TNF-α stimuli. WT or AWP1 KO MCF-7 cells were incubated with TNF-α for 48 h and then analyzed by qPCR using selective primers for E-cadherin **(H)**, and Snail, Slug, IL-6, or IL-8 **(I)**. **(J)** WT or AWP1 KO cells were pretreated with NF-κB inhibitor (NF-κBi) and the transcript levels of IL-6, Snail, Slug, and IL-8 were determined using qPCR; *p < 0.05, **p < 0.005, ***p < 0.001 versus the indicated group. N.S., not significant. All statistical comparisons were performed using t-tests (two groups) or ANOVA with a Tukey post-test (multiple groups).

**Figure 4:**
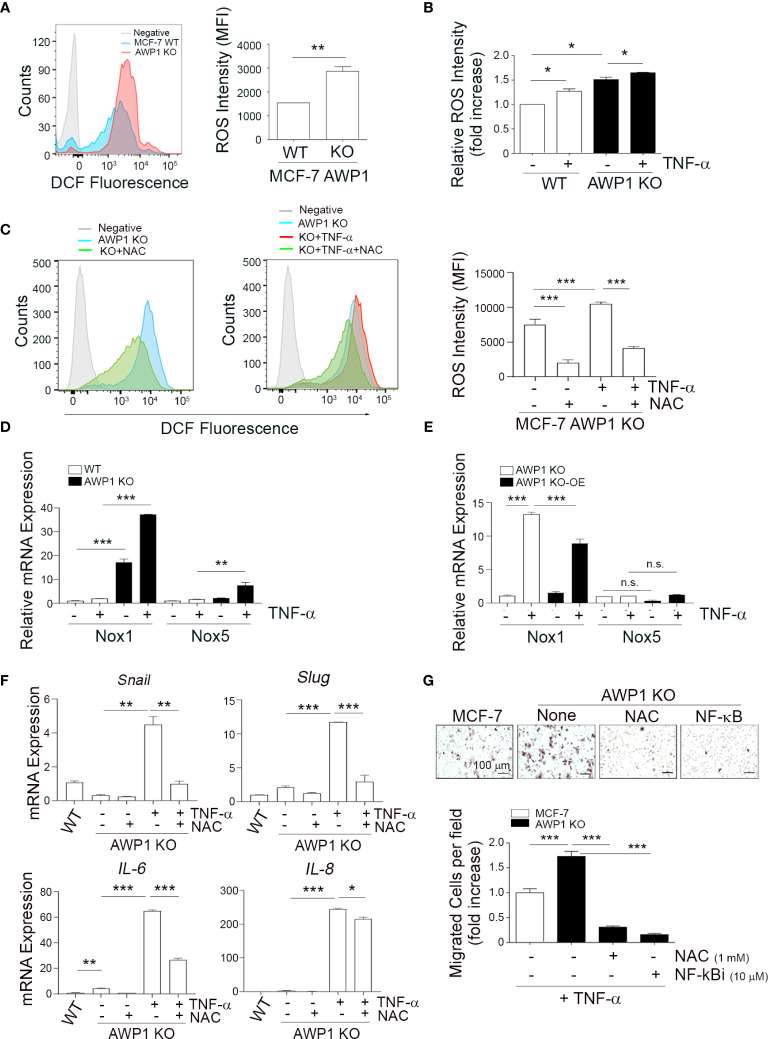
AWP1 depletion induces breast cancer cell motility *via* TNF-α-induced ROS generation. **(A)** An AWP1 deficiency increases ROS generation. Intracellular ROS levels were quantified by FACS analysis using DCF fluorescence in AWP1 WT or AWP1 KO MCF-7 cells; **p < 0.005. **(B)** TNF-α administration promotes ROS generation. AWP1 WT or AWP1 KO MCF-7 cells were treated with TNF-α and the relative ROS intensity was measured using FACS analysis. Results are expressed as the fold increase versus WT; *p < 0.05. **(C)** AWP1 WT or AWP1 KO MCF-7 cells were pretreated with N-acetyl-l-cysteine (NAC, 5 mM) and then incubated with 20 ng/ml TNF-α. The ROS intensity was measured using FACS analysis; ***p < 0.001. **(D)** WT and AWP1 KO cells were treated with TNF-α for 24h and then Nox1 and Nox5 transcript levels were determined by qPCR. **p < 0.005, ***p < 0.001 vs corresponding group. **(E)** AWP1 KO cells were infected with lentiviruses harboring a control vector (pCDH) or a AWP1 overexpression (OE) construct (pCDH-AWP1) for two days. AWP1 KO (infected with lentiviruses with pCDH) and AWP1 KO-OE (infected with lentiviruses with pCDH-AWP1) cells were incubated with TNF-α for 24h. Nox1 and Nox5 mRNA expression was estimated by qPCR analysis. **p < 0.005, ***p < 0.001 vs corresponding group. n.s., not significant. **(F)** AWP1 regulates the expression of EMT-related genes *via* ROS production in AWP1 KO cells. AWP1 KO MCF-7 cells were incubated with TNF-α for 48 h and the transcript levels of Snail, Slug, IL-6, and IL-8 were then analyzed by qPCR. For ROS inhibition, the cells were pretreated with NAC prior to TNF-α stimulation; *p < 0.05, **p < 0.005, ***p < 0.001 versus the indicated group. **(G)** An AWP1 deficiency increases the cell migratory potential of MCF-7 cells *via* TNF-α-induced ROS generation. AWP1 WT or AWP1 KO MCF-7 cells were loaded into the upper chamber of a Transwell system and stimulated with TNF-α in the lower chamber. Cells were pretreated with NAC prior to Transwell migration and the number of migrated cells were stained (*upper*) and quantified (*lower*); ***p < 0.001 vs the indicated group. All statistical comparisons were performed using t-tests (two groups) or ANOVA with a Tukey post-test (multiple groups).

The study results are presented as a mean ± SEM. All quantitative experiments were performed at least three times independently. ANOVA, a Student’s t test, and a Tukey post-test were performed using GraphPad Prism 6 (La Jolla, CA). One-way ANOVA tests were performed for two-group comparisons and the Tukey post-test was used for multiple comparisons (**Figures 1**–**4**). P values below 0.05 were considered to indicate statistical significance (*p < 0.05, **p < 0.005, ***p < 0.001).

## Results

### AWP1 Knockdown Enhances TNF-α-Induced Responses in MCF-7 Breast Cancer Cells

It has been well demonstrated that aggressive MDA-MB231 cells have higher migratory potential than non-aggressive MCF-7 breast cancer cells (10). Indeed, MDA-MB231 cells exhibit much higher migratory and proliferation capacity compared to MCF-7 cells (**Figure S1A, B**). Interestingly, immunoblotting assay revealed that AWP1 protein expression was relatively reduced in MDA-MB-231 compared to MCF-7 (**Figure S1C**), implying that the expression of AWP1 may be involved in migrative activity of breast cancer cells.

Previous studies have suggested that aggressive and non-aggressive breast cancer cells show differing susceptibilities to TNF-α-induced tumor responses, including cancer cell migration (11–13). To further examine the effects of TNF-α on the aggressiveness levels of breast cancer cells in our present analyses, we compared the cell growth rates and migratory capacity of the non-aggressive MCF-7 and aggressive MDA-MB231 breast cancer cell lines following exposure to this cytokine (20 ng/ml). The TNF-α-induced cell growth rates showed no significant differences between these cell lines (**Figure S1D**). In contrast, however, TNF-α markedly increased the migration of MDA-MB231 cells and modestly enhanced that of MCF-7 cells (**Figure 1A**).

We previously reported that AWP1 binds to TNF receptor-associated factor 2 (TRAF2), which is involved in the NF-κB signaling pathway (27) that plays a critical role in cancer cell migration. To determine the potential involvement of AWP1 in breast cancer progression in our current analyses, we first compared its expression between the MCF-7 and MDA-MB231 cell lines. MCF-7 cells expressed much higher basal levels of AWP1 transcript and protein than MDA-MB231 cells, and this difference was further increased by TNF-α treatment (**Figures 1B, C**). To next determine whether AWP1 directly affects breast cancer cell migration, the endogenous protein was knocked down in MCF-7 cells using a combination of three specific small interfering RNAs (siRNAs) targeting AWP1. The AWP1 mRNA level was effectively reduced to approximately 50% of the control level as revealed by qPCR (**Figure S1E**). We examined the effect of AWP1 knockdown on the proliferation of MCF-7 cells and observed no significant difference in cell growth between control siRNA-transfected and AWP1 siRNA-transfected MCF-7 cells, as accessed using a CCK-8 assay (**Figure S1F**). We next conducted a wound healing assay to investigate the role of AWP-1 in the migration of breast cancer cells. The AWP1-knock-downed MCF-7 cells displayed enhanced migration in comparison with the control siRNA-transfected cells (**Figure S1G**). The AWP1 knock-down using short hairpin RNA (shRNA) was verified at the protein level (**Figure 1D**). AWP1-knock-downed MCF-7 cells displayed an aggressively enhanced migration ability as revealed by a Transwell assay (**Figure 1E**). Similarly, knockdown of AWP1 in non-aggressive T47D breast cancer cells also resulted in significantly enhanced migration ability (**Figure S2**). In addition, TNF-α treatment further augmented the migratory capacity of the AWP1-silenced MCF-7 cells (**Figure 1F**). Taken together, these results suggest that AWP1 regulates TNF-α-induced migrative potential.

### A CRISPR/Cas9-Mediated Knockout of the AWP1 Gene Enhances the Migration Ability of MCF-7 Cells and Induces Morphological Changes

To knockout AWP1 in breast cancer cells using the CRISPR/Cas9 system, we first searched for all possible spCas9 target sequences within the AWP1 coding sequence (CDS). However, most of them had potential off-target sites also, which could target AWP1 pseudogene 1 (ZFAND6P1, NG_029329.2 from NCBI). To minimize these potential off-target effects, we selected two target sites at the intron-exon junctions which would not target ZFAND6P1 (**Figure 2A**). The two gRNAs were co-transfected with spCas9 into MCF-7 cells and single clones were isolated and analyzed to determine whether the AWP1 genes were disrupted. As two gRNAs were used to delete the exon 3 of NM_019006.4 variant containing the start codon, single clones with AWP1 disruption were easily detected by the size of PCR amplicons. A total of 62 single clones were analyzed and three single clones (#35, #40 and #44) were selected for expansion. The AWP1 knockout was finally confirmed by targeted deep sequencing (**Figure 2B**). We also determined the potential off-target sites with more than 3bp mismatches, in silico, and analyzed whether off-target mutations were induced in the three single clones. Among 22 potential off-target sites, the OT1-3 site was mutated in two single clones (#35, #44) with 53% and 47%, respectively, and we assumed that the two clones had heterozynous OT1-3 site induced by CRISPR/Cas9 system. The #40 single clone had no detectable mutations across any potential off-target sites (**Figure 2C** and **Table S2**).

To confirm the involvement of AWP1 in breast cancer cell migration, we selected MCF-7 cell lines of colony AWP1 KO#35 (high expression of AWP1), AWP1 KO#40 (low expression of AWP1), and AWP1 KO#44 (intermediate expression of AWP1). We observed that AWP1KO#40 displayed robustly decreased AWP1 levels compared with the parent MCF-7 cells, as confirmed by RT-PCR (**Figure 2D**, *upper*) and immunoblotting (**Figure 2D**, *lower*). Specially, AWP1 KO#40 cells underwent morphologic changes from an epithelial to a spindle-shaped phenotype (**Figure 2E**), indicating that a knockout of AWP1 alters the breast cancer cell morphology towards a mesenchymal cell type. We next examined the effects of an AWP1 deficiency on the proliferation and migration of MCF-7 cells. We observed no significant change in the cell growth of AWP1 KO#40 cells compared to MCF-7 WT cells, as shown by a clonogenic growth assay (**Figure 2F**). In comparison, the transmigration capacity was prominently increased in AWP1 KO#40 cells when compared with MCF-7 WT cells (**Figure 2G**).

### An AWP1 Knockout Induces the TNF-α-Mediated Activation of NF-κB and the Expression of Epithelial-Mesenchymal Transition-Related Genes

Given the increase in the migration of AWP1 silenced breast cancer cells in response to TNF-α (**Figure 1E**), we next evaluated the effects of an AWP1 knockout on the TNF-α-mediated migration of MCF-7 breast cancer cells. A Transwell assay revealed that the AWP1 knockout itself enhanced the migration capacity of MCF-7 cells and that TNF-α treatment further increased this migratory capacity (**Figures 3A, B**). It is known that TNF-α activates NF-κB, which is required for TNF-α-mediated gene expression, and that the IKK-mediated phosphorylation of IκBα is a key step in this activation (4). To determine whether AWP1 is involved in TNF-α-mediated phosphorylation and degradation of IκBα, AWP-1 KO and WT cells were treated with TNF-α, and IκBα protein expression was determined by immunoblotting. The knockout of AWP1 markedly increased TNF-α-induced IκBα phosphorylation and degradation compared with the WT control (**Figure 3C**). Next, we determined the expression level and localization of NF-κB/p65 utilizing immunofluorescence staining in these cells and found that AWP1 knockout led to increased NF-κB/p65 expression in both cytosol and nucleus and accumulation of this protein in nucleus (**Figure S3**). We analyzed the effects of the AWP-1 knockout on NF-κB activity using an NF-κB activity using luciferase reporter assay, which harbors tandem repeats of the NF-κB transcriptional response element. Consistent with the increased degradation of IκBα (**Figure 3C**), the AWP1 knockout led to an enhancement of NF-κB activity when compared to the WT control (**Figure 3D**), indicating that the loss of AWP1 may induce the TNF-α-mediated activation of NF-κB. In contrast, overexpression of AWP1 significantly attenuated both NF-κB activity in basal level (**Figures 3E, F**) and TNF-α-mediated migration ability in MDA-MB231 cells (**Figure 3G**). To then determine the effect of the AWP1 knockout on the TNF-α-mediated expression of EMT-related genes, WT and AWP1 KO cells were treated with or without TNF-α. The AWP1 knockout decreased E-cadherin mRNA and protein expression level (**Figures 3H**, **S4**), whilst the expression of EMT-related genes such as snail, slug, IL-6, or IL-8 was increased in AWP1 KO cells in response to TNF-α (**Figure 3I**). Moreover, the expression levels of Snail, Slug, and IL-8 proteins in AWP1 KO cells were significantly higher than those of WT cells, although IL-6 was not detectable in these cells (**Figure S5**). In comparison, the TNF-α-mediated increase in IL-6, slug, or IL-8 expression was significantly blocked in AWP1 KO cells by an NF-κB inhibitor (**Figure 3J**), reinforcing the involvement of AWP1 in NF-κB activation.

### An AWP1 Deficiency Enhances Cell Motility *via* TNF-α-Induced ROS Generation

It has been well established that ROS generation closely correlates with enhanced tumor cell motility (24, 32). To further elucidate the role of AWP1 in breast cancer cell migration in association with ROS, we examined whether the intracellular ROS levels are increased in AWP1 KO breast cancer cells, and thereby affect breast cancer cell motility. Higher levels of ROS were constitutively generated in the AWP1 KO MCF-7 cells compared to AWP1 WT MCF-7 cells, as determined by FACS analysis (**Figure 4A**) and these ROS levels were further increased by TNF-α stimulation in both cell types (**Figure 4B**). However, this effect was significantly inhibited by treatment with the ROS scavenger, N-acetyl-l-cysteine (NAC) (**Figure 4C**). Next, to further elucidate the potential involvement of AWP1 in Nox (ROS- generating enzymes)-mediated ROS generation, we compared the mRNA levels of Nox family members in WT and AWP1 KO cells followed by TNF-α stimuli. We found that the Nox1 and Nox5 mRNA levels of AWP1 KO breast cancer cell lines were significantly higher than those of WT cells and these enhancement of Nox1 and 5 were further induced by TNF-α exposure (**Figure 4D**). In addition, the ectopic expression of AWP1 in AWP1 KO cells significantly attenuated the level of Nox1 but not Nox5 (**Figure 4E**), suggesting that Nox1 may be a potential component of AWP1-mediated ROS generation.

Given that EMT plays crucial roles in cancer cell migration (33), we examined whether the TNF-α-mediated enhancement of the expression of EMT-related genes in AWP1 KO cells could be inhibited by NAC. The increases in the transcript levels of the EMT-related genes induced by TNF-α including snail, slug, IL-6, and IL-8 were indeed inhibited by NAC treatment (**Figure 4F**). Furthermore, the TNF-α-mediated increase in the chemotactic migration of AWP1 KO cells was completely abrogated by both NAC and an NF-κB inhibitor (**Figure 4G**). Taken together, these results indicated that AWP1 may act as a negative regulator of ROS-NF-kB activation in association with EMT genes linking to the cancer-promoting effects.

## Discussion

AWP1, a ubiquitin-binding NF-κB modulator, is ubiquitously expressed in a wide variety of tissues (27). Our current study findings have revealed that AWP1 plays a crucial role in the TNF-α-induced migration of breast cancer cells by showing that the ‘Nox1-ROS–NF-κB–EMT-related genes’ cascade can be potentially regulated by AWP1. In support of AWP1 as a negative regulator of breast cancer cell migration, an AWP1 deficiency alone induced morphologic changes in breast cancer cells from an epithelial phenotype towards a mesenchymal phenotype, and also induced enhanced cell motility (**Figure 2**). In addition, we observed that the CRISPR/Cas9-mediated knockout of endogenous AWP1 promoted TNF-α-induced NF-κB activation and chemotaxis in AWP1 KO cells (**Figure 3**). We conclude from our current data that AWP1 has negative effects on cancer-promotion through its regulation of NF-κB-dependent activation induced by TNF-α. Our present study results indicate that AWP1 acts as an endogenous brake towards NF-κB activation, and thus to its cancer-promoting effects, in breast cancer cells.

Consistent with our current observations, several studies have suggested that TNF-α is frequently upregulated in breast cancer and exerts diverse functions in cancer-promoting processes such as cell migration (34). Another prior study has shown that TNF-α directly and/or indirectly induces proteases such as MT1-MMP and MMP9, and also promotes CCR7 upregulation (35), thereby contributing to cancer cell migration from breast and prostate tumors. In addition, TNF-α has been found to enhance cell motility by promoting invadopodia formation in MDA-MB-231 cells (11). Gao et al., also recently demonstrated that TNF-α mediates breast cancer cell migration through YAP signal activation, indicating the involvement of both canonical and non-canonical NF-κB signaling in this process (36). Moreover, TNF-α was reported to increase the proportion of breast cancer stem-like cells through NF-κB/HIF1α/Slug, indicative of primary breast tumors with self-renewal capacities that are resistant to cell death (37). TNF-α is also characterized by an imbalance between survival and apoptosis signals in many pathological situations, including cancer (38). We show from our current analyses that TNF-α dramatically increases the AWP1 mRNA level in MCF-7 cells which display low motility and subsequently enhances their migration potential to a significant extent, but only modestly increases this potential in MDA-MB-231 cells which already display a high migratory capacity (**Figure 1C**). These findings suggested that AWP1 may function as a regulatory protein during TNF-α-induced cancer activity.

Our present experiments show that an AWP1 deficiency induces EMT gene expression and breast cancer cell migration, indicative of an aggressive tumor phenotype, which is mediated by Nox1-ROS-NF-κB-linked signal activation *via* TNF-α stimulation. We predict therefore that TNF-α may differentially regulate the AWP1 protein level in breast cancer cells and that AWP1 acts as a negative regulator of TNF-α-induced cancer promoting processes. TNF-α signaling has been associated previously with aggressive breast cancer cells (18) and in the promotion of chemotactic migration through ROS generation (21). The link between ROS and AWP1 generation in cancer cells still remains unknown, although AWP1 is expressed in various normal tissues. In our current study, the ROS levels in AWP1 KO cells were higher than in MCF-7 WT cells, and this effect were significantly promoted by TNF-α stimulation (**Figure 4**).

Previous studies have shown that TNF-α alters the redox status, and in turn activates NF-κB in cancer cells (39, 40). We previously reported that AWP1 binds TNF receptor-associated factor 2 (TRAF2) that regulates NF-κB activation under TNF-α stimulation, although the correlation between AWP1 and redox status has not been elucidated yet. Nevertheless, it has been suggested that TRAF2 is involved in ROS-induced intracellular signaling processes (41) and activates NF-κB pathway by generating ROS through TNF-α-mediated induction of cytosolic phospholipase A2 in pulmonary alveolar epithelial cells (42). We showed that AWP1 KO using Crispr-Cas9 system in MCF-7 cells induced Nox1 expression and ROS generation (**Figure 4**). Given the association of AWP1 with TRAF2 which regulates ROS generation, it is tempting to postulate that AWP1 may affect TRAF2-mediated ROS generation. However, further studies are required to determine detailed regulatory mechanisms by which AWP1 regulates ROS generation in association with TRAF2 and Nox1. ROS are also known to be closely correlated with the NF-κB pathway and, as a result, stimulate the MMPs involved in invasion and metastasis (43, 44). Consistent with our current observations shown in **Figures 3** and **4**, an increased TNF-α level in cancer cells acts as a positive autocrine feedback signal to activate NF-κB, which further induces the expression of TNF-α and other cytokines such as GM-CSF and IL-8 (45). Hence, the depletion of AWP1 may accelerate the cancer-promoting potential of cells in association with TNF-α signaling and dependent on ROS/NF-κB activation.

## Data Availability Statement

The raw data supporting the conclusions of this article will be made available by the authors, without undue reservation.

## Author Contributions

E-YK, J-EK, BC, and E-JC designed the research. E-YK, J-EK, H-SL, E-JL, JK, S-OP, HC, and HS performed the experiments. E-YK, J-EK, JK, BC, YK, and E-JC analyzed the data. E-YK, J-EK, BC, YK, and E-JC prepared the manuscript. All authors contributed to the article and approved the submitted version.

## Funding

This work was supported by a National Research Foundation of Korea (NRF) MRC grant (2018R1A5A2020732) and a grant of the Basic Science and Engineering Research Program (2018R1A2B2001867) funded by the Korean government to E-JC and a grant (NRF-2020R1I1A1A01051927) from the National Research Foundation of Korea to E-YK.

## Conflict of Interest

The authors declare that the research was conducted in the absence of any commercial or financial relationships that could be construed as a potential conflict of interest.
