# Supplementary material for: AWP1 Restrains the Aggressive Behavior of Breast Cancer Cells Induced by TNF-α
Source: Front Oncol. 2021 Mar 18;11:631469. doi: 10.3389/fonc.2021.631469 (PMC8012775; doi:10.3389/fonc.2021.631469)
Supplement: Supplementary file 1 [file DataSheet_1.docx]

**SUPPLEMENTARY INFORMATION**

**MATERIALS AND METHODS**

**Immunofluorescence staining**

WT and AWP1 KO cells were grown on coverslips in 35 mm dishes for 24 h. These cells were then washed three times with ice-cold PBS and fixed with 2% paraformaldehyde (PFA). After being blocked with 2% bovine serum albumin for 30 min, cells were incubated with the primary antibody against E-cadherin (1:100) or NF-κB/p65 (1:200 dilution) (Cell Signaling Technology Inc., MA) for 24 h at 4 ℃. Cells were then washed three times with PBS and incubated with fluorescein isocyanate-conjugated secondary antibody (1:200 dilution, Molecular Probes, Eugene, OR). The filaments of cytoplasm and nuclei were stained with phalloidin-conjugated 633 (1:200) and 2.5 μg/ml propidium iodide, respectively. All images were collected with a confocal laser scanning microscope (PerkinElmer, Waltham, MA).

**Transient transfection of small** **interfering RNA**

Negative control small interfering RNA (siRNA) oligonucleotides and siRNA oligonucleotides targeting human AWP1 were obtained from OriGene Technologies (Rockville, MD). MCF-7 cells were seeded in 6-well plates at a quantity of 1 × 10^5^ cells and transfected with siRNA against AWP1 or negative control siRNA using Lipofectamine™ RNAiMAX (Invitrogen, Merelbeke, Belgium) according to the manufacturer’s instructions. The cells were analyzed 36h post-transfection and the level of AWP1 mRNA was evaluated by qPCR.

**IL-6 ELISA**

1 x 10^5^ cells of AWP1 WT or KO cells were cultured in 6-well plates and additionally incubated with/without TNF-α for 48h. The supernatants of cells were harvested and the level of IL-6 protein was measured using a IL-6 specific sandwich ELISA kit (BioLegend, San Diego, CA) in accordance with the manufacturer’s protocols. All samples were examined in triplicate for each experiment.

**Supplementary Table 1. PCR primers used in this study**

| Gene name | Primer sequence | |
| --- | --- | --- |
| human AWP1 | Forward | 5'-ATGGCTCAAGAAACTAATCACA-3' |
|  | Reverse | 5'-CTTCTGGCACACTGCCATCTGT-3’ |
| human Snail | Forward | 5’-ACTGCAACAAGGAATACCTCAG-3’ |
|  | Reverse | 5’-GCACTGGTACTTCTTGACATCTG-3’ |
| human Slug | Forward | 5’-AAGCATTTCAACGCCTCCAAA-3’ |
|  | Reverse | 5’-GGATCTCTGGTTGTGGTATGACA-3’ |
| human cdh1 | Forward | 5’-CGGTGGTCAAAGAGCCCTTACT-3’ |
|  | Reverse | 5’-TGAGGGTTGGTGCAACGTCGTTA-3’ |
| human IL-6 | Forward | 5’-TTAGAGTCTCAACCCCCAATAAATA-3’ |
|  | Reverse | 5’-GTGGGGCGGCTACATCTTT-3’ |
| human IL-8 | Forward | 5’-TTTTGCCAAGGAGTGCTAAAGA-3’ |
|  | Reverse | 5’-AACCCTCTGCACCCAGTTTTC-3’ |
| human Nox1 | Forward | 5'-CTG CTT CCT GTG TGT CGC AA-3’ |
|  | Reverse | 5'-AGG CAG ATC ATA TAG GCC ACC-3’ |
| human Nox5 | Forward | 5'-GGC TCA AGT CCT ACC ACT GGA-3’ |
|  | Reverse | 5'-GAA CCG TGT ACC CAG CCA AT-3’ |
| human GAPDH | Forward | 5′-TGTTGCCATCAATGACCCCTT-3′ |
|  | Reverse | 5′-CTCCACGACGTACTCAGCG-3′ |

**Supplementary Table 2. Analysis of mutation frequencies of the potential off-target sites of two ZFAND6 targeting gRNAs**


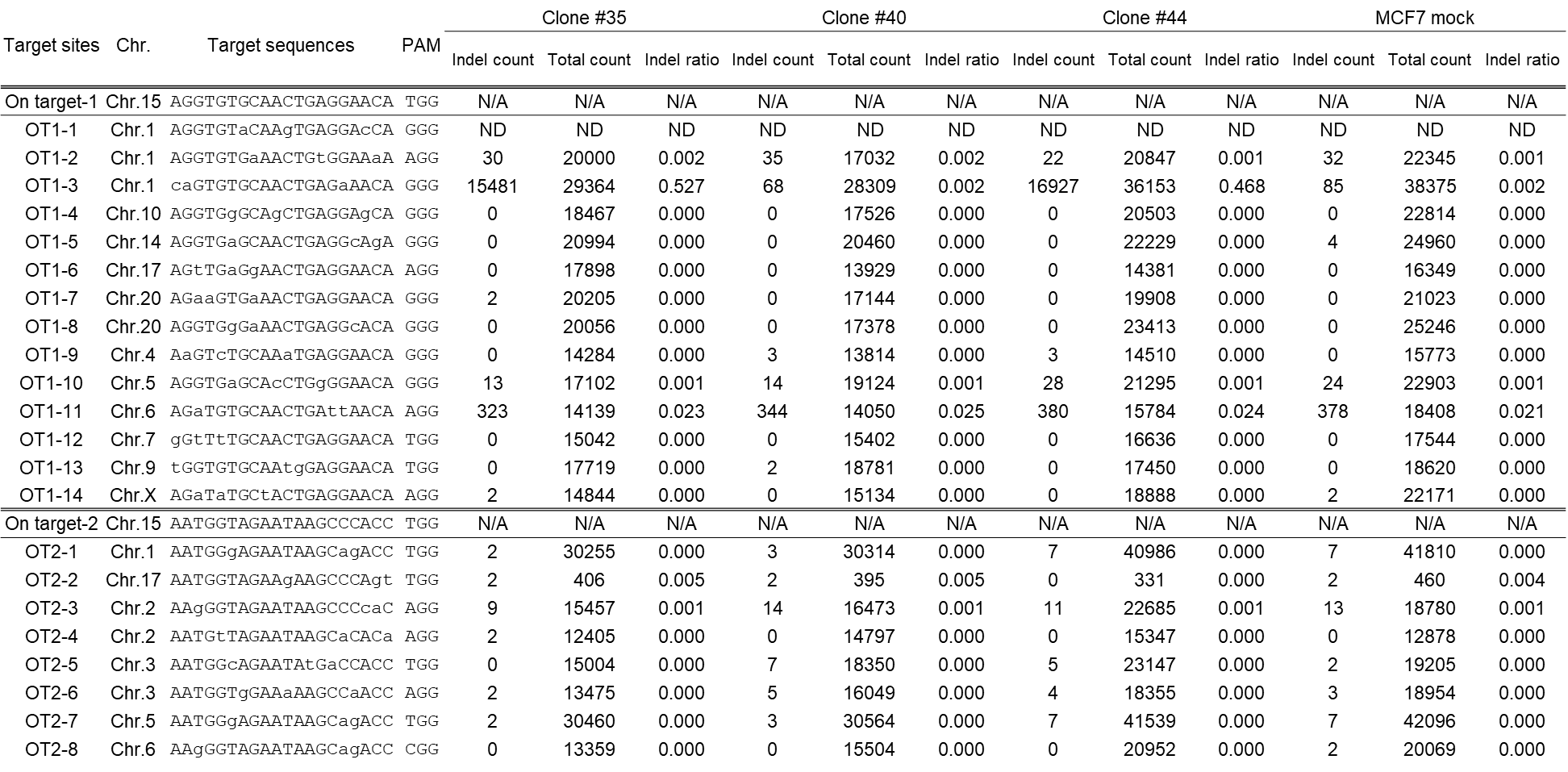


**Supplementary** **Table 3. Primer list used for target**


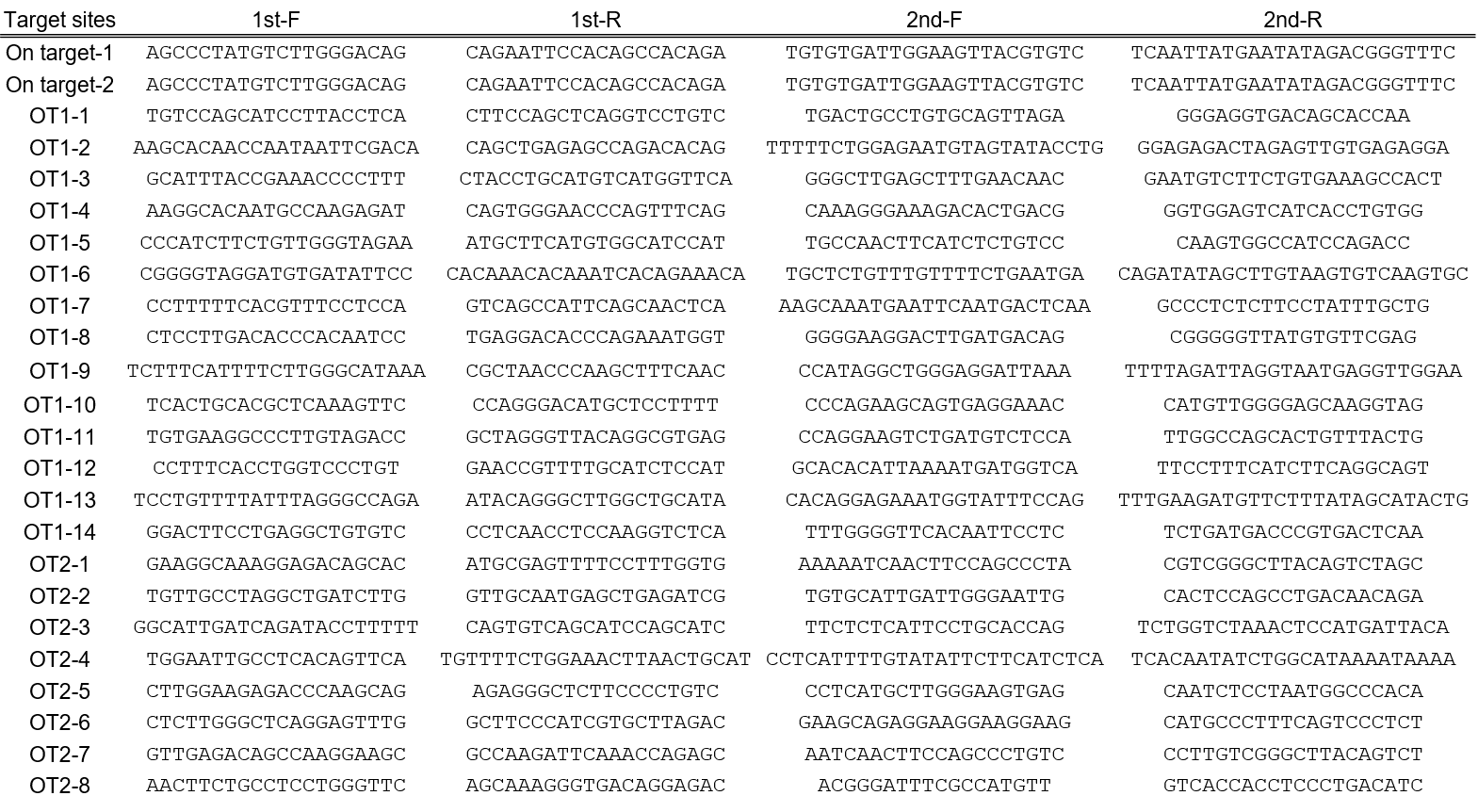


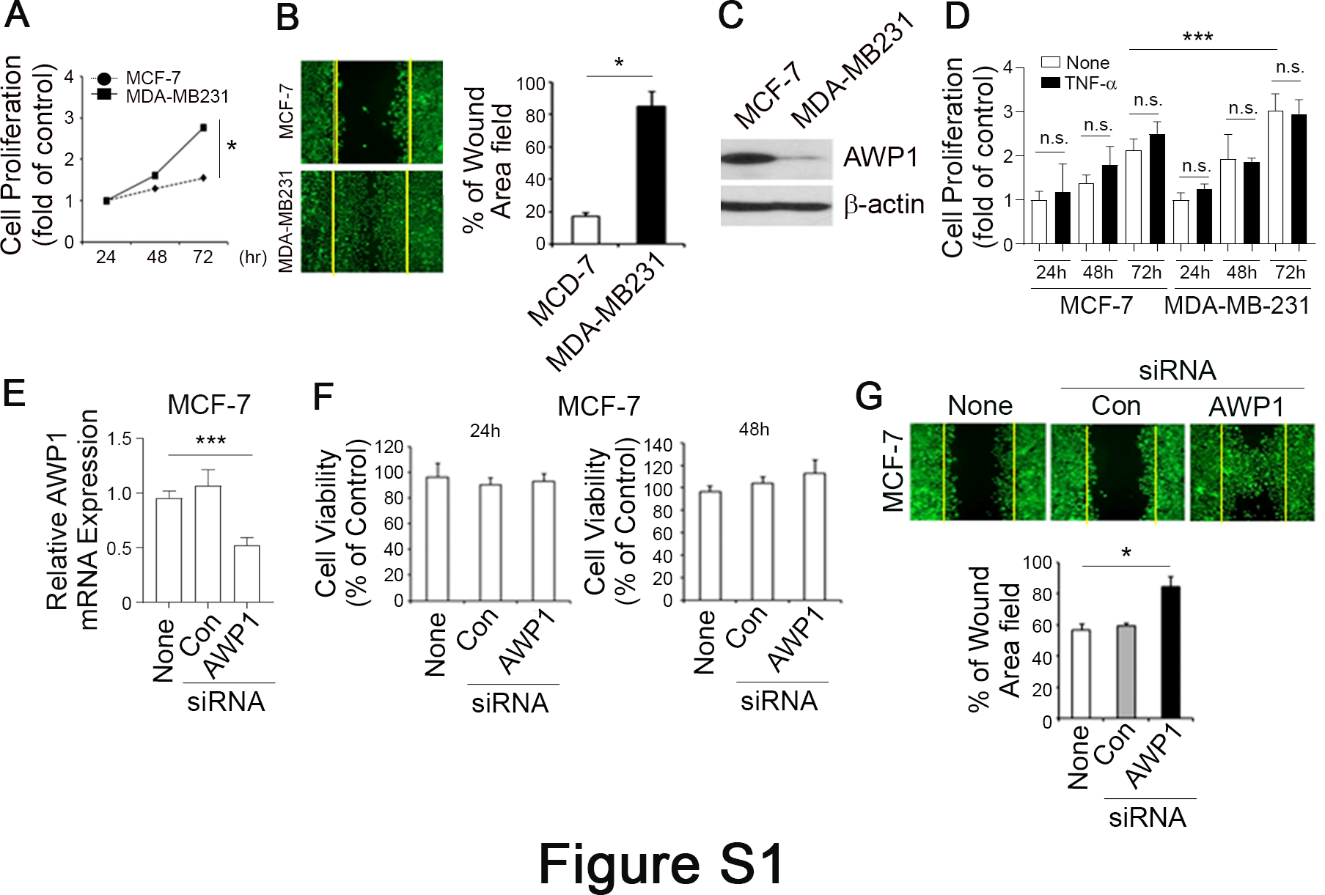


**FIGURE S1.** AWP1 knockdown increases migratory potential of human breast cancer cells. **(A-C)** Proliferation rate **(A)** and wound-healing migration ability **(B)** of MCF-7 and MDA-MB231 breast cancer cells were determined. **(C)** AWP1 protein expression level in these cells was determined by immunoblotting. **(D)** The growth rate of MDA-MB231 and MCF-7 cells in response to TNF-α was estimated by CCK-8 assay for 24, 48, or 72 h. n.s., not significant. **p* < 0.05, ****p* < 0.001 corresponding to MCF-7 cells by Tukey’s test. **(E)** MCF-7 cells were transfected with control siRNA or AWP1-specific siRNA and AWP1 knockdown was confirmed using qPCR. **(F)** Cell viability of MCF-7 cells transfected with control siRNA or AWP1-specific siRNA was measured using CCK-8 assay kit. **(G)** Silencing of AWP1 increases motility ability of MCF-7 breast cancer cells. Wound healing assay was performed to investigate the role of AWP-1 in the migration of breast cancer cells. * *p* < 0.05 compared to siRNA control by t-tests.


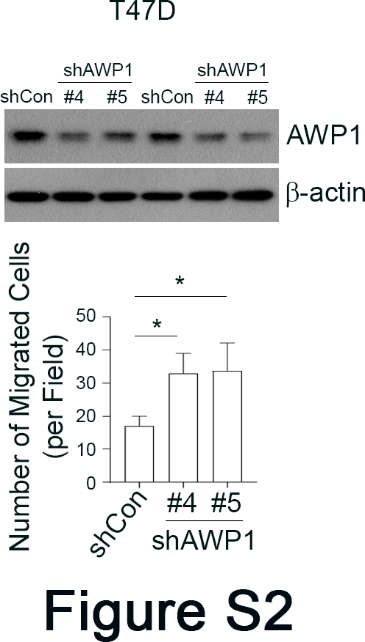


**FIGURE S2.** AWP1 knockdown increases migratory potential of T47D breast cancer cells. T47D cells were infected with control shRNA or AWP1-specific shRNA retroviral particles (#4 and #5). Silencing of AWP1 by a specific AWP1 shRNA in T47D cells were confirmed by western blot (*upper*) and the migration capacity was evaluated using transwell migration assay at 24h after transfection (*lower*). * *p* < 0.05 versus control shRNA.


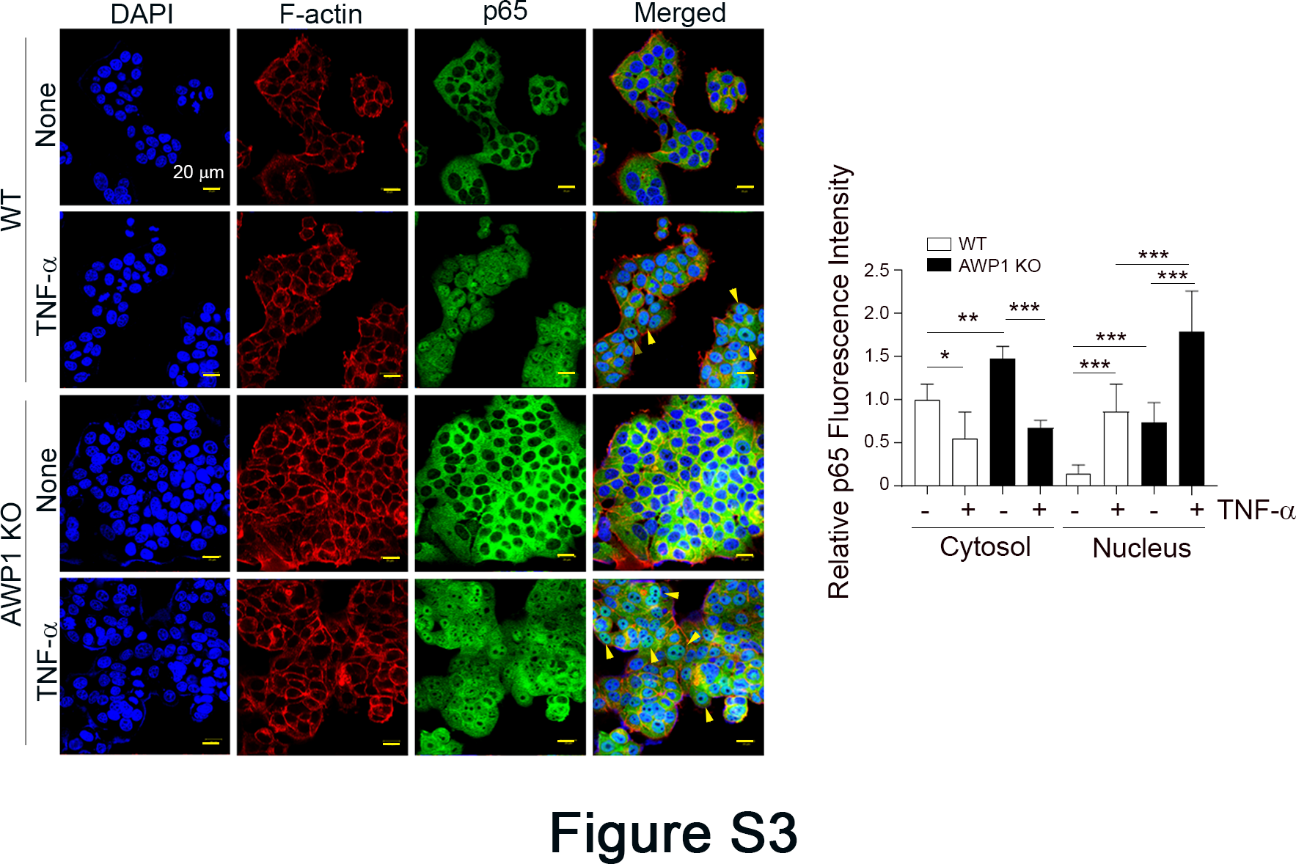


**FIGURE S3.** Deficiency of AWP1 promotes TNF-α-mediated NF-κB translocation to nucleus. WT or AWP1 KO cells were plated on the cover slides and incubated with TNF-α for 15 min. Then these cells were further analyzed by immunofluorescence staining using anti-NF-kBp65 and FITC-labeled anti-rabbit antibodies. Phalloidin F-actin and DAPI was used for staining the filaments of cytoplasm and nucleus, respectively. The arrowheads indicate NF-kBp65 localized in the nucleus. The mean intensity levels of NF-kB conjugated to FITC were quantitated in the cytoplasm and nucleus, and plotted as mean intensity±SEM. **p* < 0.05, ***p* < 0.005, or ****p* < 0.001 vs corresponding group.


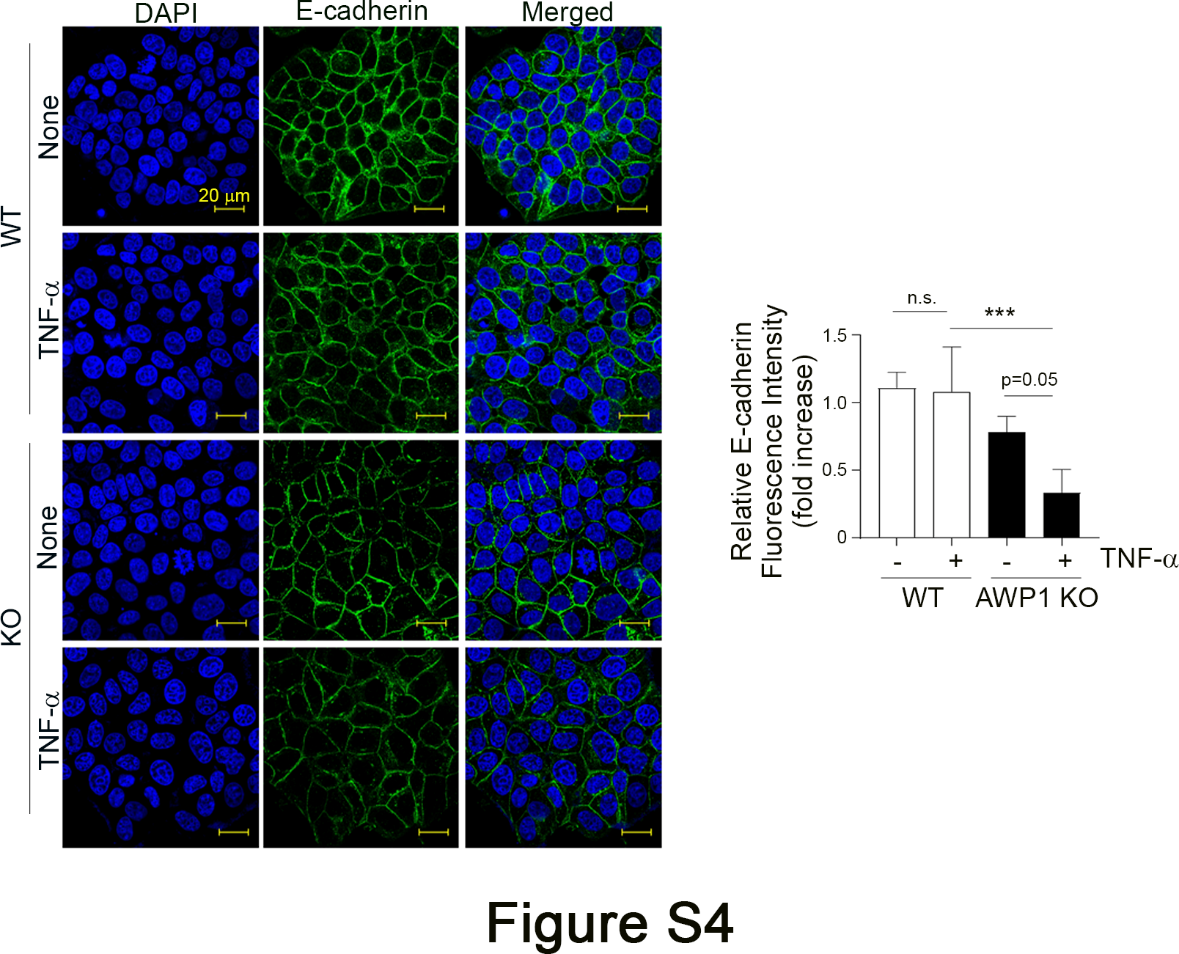


**FIGURE S4.** Depletion of AWP1 reduces E-cadherin expression. WT or AWP1 KO cells were plated on the cover slides and incubated with TNF-α for 24h. These cells were then further analyzed by immunofluorescence staining using anti-E-cadherin and FITC-labeled anti-rabbit antibodies. DAPI was used for staining the of nucleus. Results shown are representative of two independent experiments with similar results (*left*). Quantification of the E-cadherin fluorescence intensity is represented. Data are presented as the mean ± standard deviation from three independent experiments (*right*). Comparisons were performed using ANOVA (multiple groups). n.s., not significant. ****p* < 0.001 vs corresponding group.


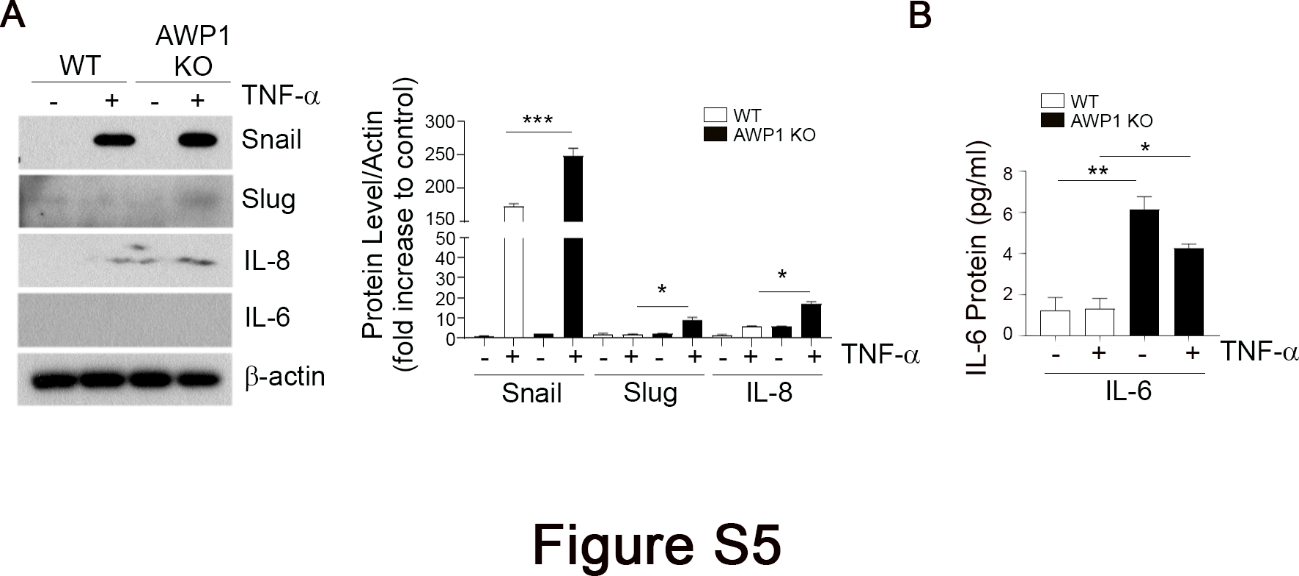


**FIGURE S5.** Depletion of AWP1 enhances Snail and IL-8 expression. **(A)** AWP1 WT and KO cells were incubated with or without 20 ng/ml TNF-α for 24 h. The protein expression levels of Snail, Slug, IL-8, and IL-6 proteins were determined by western blotting (*left*). Western blot was quantified and expressed as the ratio of proteins and ß-actin intensity (*right*). **(B)** AWP1 WT and KO cells were further incubated with or without TNF-α for 48 h. Supernatants were harvested and the levels of IL-6 protein was measured by IL-6 ELISA kit. **p* < 0.05, ***p* < 0.005, or ****p* < 0.001 vs corresponding group.
